# Supplementary material for: Learning curve and surgical outcome of robotic assisted colorectal surgery with ERAS program
Source: Sci Rep. 2022 Nov 29;12:20566. doi: 10.1038/s41598-022-24665-w (PMC9709162; doi:10.1038/s41598-022-24665-w)
Supplement: Supplementary file 1 — Supplementary Information 1. [file 41598_2022_24665_MOESM1_ESM.zip › LSC Raw data-Ñ[▒K/097/N_ñw╝╨░O▒Kñσ.pdf]

W52 / W52

臺 中 榮 民 總 醫 院
手術室護理記錄

[redacted]

基本資料

- 室別： 開刀房-06室，手術科別： CRS，手術類別： 常規，日期： 2020/10/15
- 進入時間： 0755 ； 麻醉開始時間： 0800 ； Time Out時間： 0917 ； 劃刀時間： 0918 ； 手術結束時間： 1320 送出時間： 1330 ；
- 麻醉方式： GE
  診斷：Malignant tumor of anal canal and anus
  手術名稱：1.Abdominoperineal resection for neogrowth of anorectum (APR)2.Laparoscopy
  手術局部用藥：nil
  術前預防性抗生素：
  - 抗生素名稱一： Veterin 1GM IVD/由麻醉護理師執行
  - 時間： 0855
  - 上次簽章: 陳秋誼

  ，追加預防性抗生素：
- 手術醫師： 蔣鋒帆，陳瑢夫，楊捷儒
- 麻醉醫師： VS 王賜洲
-
  - 刷手護士A ： 陳映庭，時間： 0755，接替刷手一： 陳秋誼，時間： 1230
  - 流動護士A ： 陳秋誼，時間： 0755，接替流動一： 戴培雅，時間： 1200

皮膚狀況

A-V Shunt： 無
術前： 完整
　記錄者： 上次簽章: 陳秋誼
術後： 完整
　記錄者： 上次簽章: 戴培雅

手術準備

手術臥式 單一手術臥式

-
  - 手術臥式： 截石臥式
  - 使用附件： 溫毯 38 ℃，矽膠枕頭，腳架，其它： 頭架
  - 皮膚準備： 10%Aq Betadine，Hibiscrub，2% Chlorhexidine
  - 電燒： 使用電燒: 電極片位置： 左大腿，其它： 迴路電擊墊，記錄者： 上次簽章: 陳秋誼

止血帶T： 無

植入物

- 植入物名稱/號碼/數量/部位：Ethicon endo GIA 60m-4.1mm x1 /at colon

，記錄者：上次簽章: 戴培雅

標本送檢

- 細菌培養 無
- 細胞學 無
- 冰凍切片 無
- 病理標本
  組織名稱一：Rectum
  ,
  組織名稱二：Pelvis
- 截肢 無

標本交付 標本： 2 袋，標本： 0 盒，標本： 0 瓶，細胞學： 0 管，Culture Tube： 0 管，被交付醫師： R 楊捷儒，

- 記錄者： 上次簽章: 戴培雅

- 時間： 1200

傷口術後數料

傷口數料： 傷口數料： 無

- 請於圖上標示部位：

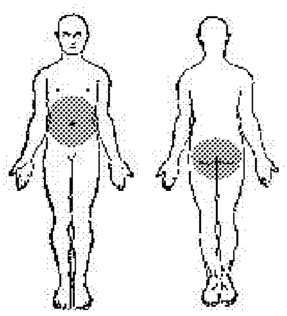

腹部五處傷口及會陰部

- 傷口數料： 腹部：menpore
  會陰部：散紗+棉墊+網褲

導管

導管	天數	導管資訊	插入日期	插入時間	更換日期	拔除日期	拔除時間	拔除原因	預計拔除/更換	預計拔除/更換日期
Penrose Drain(1)	1	號碼:12 mm.; 部位:會陰部;	2020/10/15	1115						
Epidural PCA(1)	1	部位:脊髓;	2020/10/15	0820						
Foley(1)	1	號碼:16 Fr.; 部位:膀胱;	2020/10/15	0855						
Penrose Drain(2)	1	號碼:12 mm.; 部位:會陰部;	2020/10/15	1115						
Double Lumen CVC(1)	1	號碼:8 Fr; 深度:fix 13 cm; 部位:jugular (右); 象限:RU;	2020/10/15	0850						
A-line (1)	1	號碼:20 G; 部位:左手;	2020/10/15	0835						
Sump NG Tube(1)	1	號碼:16 Fr.; 部位:胃;	2020/10/15	0905						

用物計數

- 手術中出血量： 250 c.c.
- 手術中尿量： 150 c.c.
-
  - 其它： 無

用物計數：

- 部位一： 腹部，1.器械： 正確，2.縫針： 正確，3.刀片： 正確，4.紗布： 正確，5.腹部墊： 未使用，6.紗球： 未使用，7.PEANUTS： 未使用，8.紗條： 未使用，9.絲帶： 未使用，10.針頭： 未使用，11.Vessel loop： 未使用，12.Boots： 未使用，13.Jelco： 未使用，14.棉條： 未使用，
  - 第一次：
    - 刷手護士 ： 陳映庭
    - 流動護士： 上次簽章: 陳秋誼
    - 時間： 1140

    ,
  - 第二次：
    - 刷手護士 ： 陳映庭
    - 流動護士： 上次簽章: 陳秋誼
    - 時間： 1150

    ,
  - 第三次：
    - 刷手護士 ： 陳映庭

        - 流動護士： 上次簽章: 戴培雅
        - 時間： 1200
- 部位二： 會陰部，1.器械： 正確，2.縫針： 正確，3.刀片： 正確，4.紗布： 正確，5.腹部墊： 未使用，6.紗球： 未使用，7.PEANUTS： 未使用，8.紗條： 未使用，9.絲帶： 未使用，10.針頭： 未使用，11.Vessel loop： 未使用，12.Boots： 未使用，13.Jelco： 未使用，14.棉條： 未使用，
    - 第一次：
        - 刷手護士 ： 陳映庭
        - 流動護士： 上次簽章: 陳秋誼
        - 時間： 1120

  ,
    - 第二次：
        - 刷手護士 ： 陳映庭
        - 流動護士： 上次簽章: 陳秋誼
        - 時間： 1130

  ,
    - 第三次：
        - 刷手護士 ： 陳映庭
        - 流動護士： 上次簽章: 陳秋誼
        - 時間： 1140
-
    - 第一次：
        - 流動護士：

  ,
    - 第二次：
        - 流動護士：

  ,
    - 第三次：
        - 流動護士：

備註

備註： CRS 腹腔鏡6號

- 記錄者： 戴培雅 上次簽章: 陳秋誼
- 時間： 1330

填寫護理人員 戴培雅

正式摘要

W52 / W52

doctorRank:B
